# Response bias to a randomised controlled trial of a lifestyle intervention in people at high risk of cardiovascular disease: a cross-sectional analysis

**DOI:** 10.1186/s12889-018-5939-y

**Published:** 2018-09-04

**Authors:** Adam Bayley, Daniel Stahl, Mark Ashworth, Derek G. Cook, Peter H. Whincup, Janet Treasure, Anne Greenough, Katie Ridge, Kirsty Winkley, Khalida Ismail

**Affiliations:** 1 Department of Psychological Medicine, Institute of Psychiatry, Psychology and Neuroscience, King’s College Londonz, 10 Cutcombe Road, London, SE5 9RJ UK; 2 Department of Biostatistics, Institute of Psychiatry, King’s College London, 16 De Crespigny Park, London, SE5 8AF UK; 3 Department of Primary Care and Public Health Sciences, King’s College London, Addison House, Guy’s Campus, London, SE1 1UL UK; 4 Population Health Research Institute, St George’s, University of London, Cranmer Terrace, London, SW17 0RE UK; 5 Department of Health Services and Population Research, Institute of Psychiatry, King’s College London, 16 De Crespigny Park, London, SE5 8AF UK; 6 Division of Asthma, Allergy and Lung Biology, King’s College London, Guy’s Hospital, London, SE1 9RT UK; 7 MRC & Asthma UK Centre for Allergic Mechanisms in Asthma, London, UK; 8 Department of Women and Children’s Health, School of Life Course Sciences, Faculty of Life Sciences and Medicine, King’s College London, London, UK

**Keywords:** Cardiovascular disease, Physical activity, Lifestyle, Complex intervention, Participation bias, Primary care

## Abstract

**Background:**

Research evaluating lifestyle interventions for prevention of cardiovascular disease (CVD) may not reach those most at risk. We compared the response rate to a randomised controlled trial (RCT) of a lifestyle intervention by CVD risk, ethnicity and level of deprivation.

**Methods:**

Primary care patients with a QRisk2 score ≥ 20% were invited to participate in a RCT of an intensive lifestyle intervention versus usual care. This cross-sectional analysis compares anonymised data of responders and non-responders with multiple logistic regression, using adjusted odds ratios (AORs) for QRisk2 score, ethnicity, Index of Multiple Deprivation (IMD 2010) quintile, age and sex.

**Results:**

From 60 general practices, 8902 patients were invited and 1489 responded. The mean age was 67.3 years and 21.0% were female. Of all patients invited, 69.9% were of white ethnic background, 13.9% ethnic minority backgrounds and 16.2% had no ethnicity data recorded in their medical records. Likelihood of response decreased as QRisk2 score increased (AOR 0.82 per 5 percentage points, 95% CI 0.77–0.88). Black African or Caribbean patients (AOR 0.67; 95% CI 0.45–0.98) and those with missing ethnicity data (AOR 0.55; 95% CI 0.46–0.66) were less likely to respond compared to participants of white ethnicity, but there was no difference in the response rates between south Asian and white ethnicity (AOR 1.08; 95% CI 0.84–1.38). Patients residing in the fourth (AOR 0.70; 95% CI 0.56–0.87) and fifth (AOR 0.52; 95% CI 0.40–0.68) most deprived IMD quintile were less likely to respond compared to the least deprived quintile.

**Conclusions:**

Evaluations of interventions intended for those at high risk of CVD may fail to reach those at highest risk. Hard to reach patient groups may require different recruitment strategies to maximise participation in future trials. Improvements in primary care ethnicity data recording is required to aid understanding of how successfully study samples represent the target population.

**Trial registration:**

ISRCTN, ISRCTN84864870. Registered 15 May 2012, 10.1186/ISRCTN84864870.

**Electronic supplementary material:**

The online version of this article (10.1186/s12889-018-5939-y) contains supplementary material, which is available to authorized users.

## Background

Cardiovascular disease (CVD) is the most common cause of mortality in developed nations [1]. Modifiable risk factors for CVD include tobacco use, physical inactivity, obesity and raised low-density lipoprotein (LDL) cholesterol [2–4]. Those most at risk are older males, of south Asian ethnic background, with lower educational attainment and lower socioeconomic status [5, 6]. Lifestyle intervention trials for primary prevention of CVD are a research priority [7–9], but previous trials have reported low participation rates [10, 11].

Factors associated with increased participation in intervention trials to increase walking and physical activity include white ethnicity, living in more affluent areas, middle age, female sex and university education [11, 12], although methodological factors can lead to different participation biases across trials. Whilst some previous trials have observed that those of poorest health are the most likely to respond [11, 13–15], others report that participants are healthier and more active than non-participants [10, 14, 16, 17]. Participation of ethnic minorities is important as they are at higher risk for CVD and type 2 diabetes [18]. Failure to recruit subjects at highest risk of disease may limit representativeness, underestimate effect sizes, and could lead to the implementation of interventions which increase rather than decrease health inequalities. Yet, there are few opportunities to study participation biases as those who do not respond to invitations to participate typically have not given consent to medical data access [19].

In this study we tested the hypothesis that people who have a lower risk of CVD are more likely to respond to an invite to participate in a randomised controlled trial (RCT) evaluating an intensive lifestyle intervention for reducing weight and increasing physical activity. We also tested whether potential sociodemographic markers of response such as white ethnicity and living in more affluent areas corresponded to a greater likelihood of response.

## Methods

### Setting and design

We used a cross-sectional design. The sample was derived from the target population invited for eligibility screening to participate in a RCT assessing the effectiveness of an enhanced MOtiVational intErviewing InTervention (MOVE IT) for reducing weight and increasing physical activity in people at high risk of CVD. MOVE IT compares the effectiveness of motivational interviewing (MI) and cognitive behaviour therapy (CBT) behaviour change techniques in group, individual and usual care arms. The study population consisted of patients at high risk of CVD in primary care from 12 south London Clinical Commissioning Groups (CCGs) representing an ethnically-diverse resident population of about 3 million [20]. Further details are described in the trial protocol [21]. Ethical approval for the MOVE IT trial was granted by the Dulwich ethics committee (12/LO/0917), including permission to extract anonymised data for all patients invited to participate. Data were not extracted if the patient record included an informed dissent code, indicating that patient data should not be shared with a third party.

### Participants and case definition

Participating practices screened primary care databases for eligible patients using either EMIS (EMIS Health, Leeds, UK) or Vision (In Practice Systems, London, UK) medical records systems, two of the clinical software programmes most commonly used in UK primary care. The risk of CVD was calculated using QRisk2 (QResearch, Nottingham, UK), a validated predictive tool for identifying the percentage risk of having a fatal or non-fatal cardiovascular event in the next 10 years [6]. Registered patients with a QRisk2 score estimated on medical records to be ≥20% and aged 40–74 years were invited to participate via a standardised letter from their general practitioner (see Additional file 1) which also included a participant information sheet (see Additional file 2). Patients were given a choice of response methods: either returning a reply slip in a stamped and addressed envelope following which a research assistant would telephone to arrange an appointment time, or to telephone the research team directly. Patients were excluded from the invitation if their medical records included a Read code (a coded thesaurus of clinical terms used in UK primary care databases) for past diagnosis of CVD, diabetes, kidney disease, chronic obstructive pulmonary disease, disabling neurological disorder, severe mental illness, registered blind or housebound, currently pregnant, advanced cancer or a body mass index > 50 kg/m^2^.

### Measures

The measures collected anonymously for patients invited to participate were QRisk2 score, ethnicity, postcode, age (at time of screening) and sex. QRisk2 score was estimated on medical records via a batch calculator which uses an algorithm of risk factors [6]. The variables used by the QRisk2 algorithm are age, sex, ethnicity, deprivation calculated from postcode data, smoking status, diabetes status, rheumatoid arthritis status, chronic kidney disease status, atrial fibrillation status, hypertensive treatment status, family history of CVD, body mass index (BMI), systolic blood pressure and the ratio of high-density lipoprotein cholesterol to total cholesterol. The algorithm uses age- and sex-based national averages for missing data values.

Self-report ethnicity data on medical records includes a wide variety of categories, some of which are not clearly defined. Where possible, data were grouped into white, black African or Caribbean, south Asian, other Asian, other/mixed or missing. South Asian and other Asian are coded separately as a higher CVD risk is associated with south Asian ethnicity [6]. The category other/mixed incorporates any ethnicity which is reported and does not fit into the previous categories, as well as indication of mixed ethnic background.

Postcode data were collected in order to calculate Index of Multiple Deprivation 2010 (IMD 2010; based on Lower Layer Super Output Area (LSOA)) [22]. The IMD 2010 incorporates seven domains: income deprivation, employment deprivation, health deprivation and disability, education deprivation, crime, barriers to housing and services, and living environment. All small areas in England are ranked, from 1 (most deprived) to 32,482, and data can be grouped into quintiles.

### Statistical analysis

Data are summarised as means and standard deviations (SD), or as percentages. The median IMD ranks are compared between responders and non-responders. In the unadjusted model, the odds of response were calculated for each explanatory variable separately. In the adjusted model, the odds of response were calculated via logistic regression adjusting for potential confounding by QRisk2 score, ethnicity, IMD quintile, age and sex in Stata version 14 (StataCorp, Texas, USA). General practice was included as a random effect in the model to allow for clustering by practice. Adjusted odds ratios (AORs) for age are presented per 5 year increase and for QRisk2 scores per 5 percentage point increase to provide a better comparison of the strength of the relationships.

## Results

### General practice and participant recruitment

**Fig. 1:**
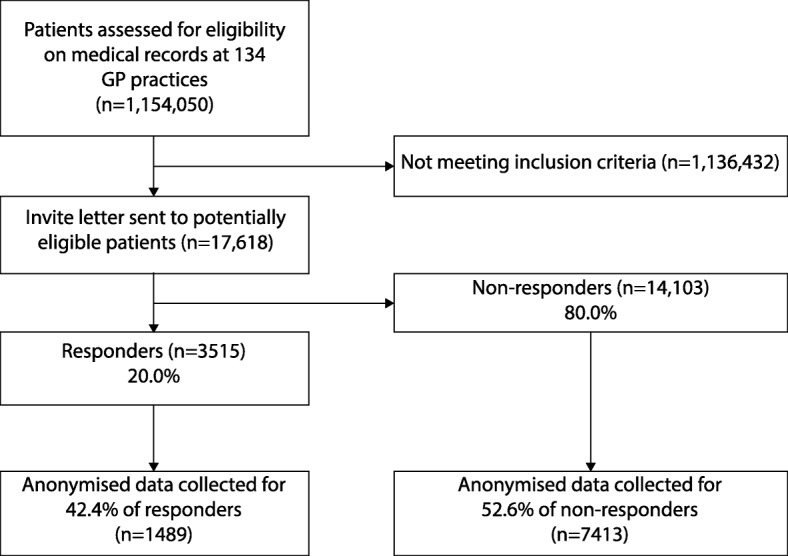
Patient response and anonymised data collection flow chart

We invited all 302 general practices with a patient list size of 5000 or more in the 12 south London CCGs, and 9 general practices with a < 5000 patient list size recruited to increase participation; 134 general practices agreed to participate and were recruited between June 2013 and December 2014. The medical records data of 1,154,050 patients were screened for eligibility and 17,618 patients were potentially eligible and invited to screening for eligibility, representing the target study population of whom 3515 patients responded (response rate 20.0%). See Fig. 1 for the patient response and anonymised data collection flow chart.

We returned to general practices to extract anonymous data following the requirements of ethical approval. The original list of potentially eligible patients who had been invited to participate had been deleted from primary care databases at many general practices; 60 practices were still able to provide the original anonymised data totaling 8902 patients (50.5% of the target population). No exclusions of data were made based on informed dissent for data sharing with a third party as no informed dissent codes were identified during the search and data extraction. Socio-demographic data and QRisk2 scores were extracted. Table 1 shows the CCGs from which anonymised data were collected and the deprivation and ethnicity Census data of each local authority [20]. General practice deprivation varies markedly within and between the CCGs, but population-weighted general practice deprivation scores (IMD 2010) of those practices from which anonymised data were extracted did not differ significantly from that of all other practices in south London (t (440) = 0.57, *p* = 0.57) [23].

**Table 1:** GP practice recruitment and data collection by Clinical Commissioning Group

	Clinical Commissioning Group											
	Bexley	Bromley	Croydon	Greenwich	Kingston	Lambeth	Lewisham	Merton	Richmond	Southwark	Sutton	Wandsworth
Total GP practices recruited	8	22	14	14	6	9	16	10	5	16	8	6
EMIS practices	1	22	13	2	2	9	16	9	0	16	7	6
Vision practices	7	0	1	12	4	0	0	1	5	0	1	0
Borough deprivation (IMD 2010 rank)^a^	180	217	99	19	252	14	16	208	286	25	193	102
Borough ethnicity (%)^b^												
White	81.9	84.3	55.2	62.5	74.5	57.1	53.6	64.9	85.9	54.3	78.6	71.4
Black African or Caribbean	8.5	6.0	20.2	19.1	2.4	25.9	27.2	10.4	1.5	26.8	4.8	10.6
South Asian	3.6	2.7	10.5	4.7	6.5	3.3	2.8	8.9	3.9	4.0	5.4	6.5
Other Asian	2.9	2.5	5.9	7.0	9.9	3.5	6.5	9.2	3.4	5.5	6.2	4.4
Other/Mixed	3.0	4.5	8.4	6.7	6.6	10.1	10.0	6.5	5.2	9.4	5.0	7.1
Anonymous data collected												
GP practices	1	11	11	1	1	6	11	2	0	9	3	4
Patients	328	2463	1663	255	27	845	1281	41	0	666	786	646

*IMD* Index of Multiple Deprivation

Data presented is *n* unless otherwise stated

^a^Ranked from 1 (most deprived) to 326 (least deprived) from 326 Local Authorities

^b^Based on CENSUS 2011 data^20^

### Participation biases

The QRisk2 and sociodemographic data of responders (*n* = 1489) and non-responders (*n* = 7413), and the results of the adjusted logistic regression, are presented in Table 2. The mean QRisk2 score of all patients invited to participate was 25.2%, and 69.9% were of white ethnic background (13.9% were of non-white ethnic background and 16.2% had no ethnicity data recorded). The median (interquartile range) IMD 2010 rank for all patients was 13,489 (7618–22,804). The mean age of all patients at the time of invite was 67.3 (5.7) years, and 20.7% were female.

**Table 2:** Comparison of responders and non-responders to trial invite

	All invited *n*=8902^a^	Responded to invitation *n* = 1489	Did not respond to invitation *n* = 7413	Response rate (%)	Unadjusted OR for response to mailout (95% CI)	Adjusted OR for response to mailout (95% CI)	Test for trend (*p*)
Age at invitation (years)	67.3 (5.7)	68.1 (5.1)	67.2 (5.8)		1.14 (1.08, 1.21)^b^	1.19 (1.12, 1.26)^b^	< 0.001
Age at invitation							
40-59 yrs	976 (11.0%)	124 (8.4%)	852 (11.6%)	12.7			
60-64 yrs	1462 (16.4%)	200 (13.5%)	1262 (17.0%)	13.7			
65-69 yrs	2989 (33.6%)	536 (36.0%)	2453 (33.1%)	17.9			
70-75 yrs	3475 (39.0%)	629 (42.2%)	2846 (38.4%)	18.1			
Sex							
Female	1847 (20.7%)	291 (19.5%)	1556 (21.0%)	15.8	1.00	1.00	
Male	7055 (79.3%)	1198 (80.5%)	5857 (79.0%)	17.0	1.07 (0.93, 1.23)	1.25 (1.08, 1.45)	0.004
Ethnicity							< 0.001^c^
White	6223 (69.9%)	1128 (75.8%)	5095 (68.7%)	18.1	1.00	1.00	
Black African or Caribbean	272 (3.1%)	34 (2.3%)	238 (3.2%)	12.5	0.74 (0.50, 1.07)	0.67 (0.45, 0.98)	0.040
South Asian	578 (6.5%)	98 (6.6%)	480 (6.5%)	17.0	0.90 (0.70, 1.14)	1.08 (0.84, 1.38)	0.542
Other Asian	240 (2.7%)	31 (2.1%)	209 (2.8%)	12.9	0.69 (0.47, 1.03)	0.69 (0.46, 1.02)	0.066
Other/Mixed	147 (1.7%)	16 (1.1%)	131 (1.8%)	10.9	0.62 (0.36, 1.05)	0.61 (0.36, 1.04)	0.072
Missing	1442 (16.2%)	182 (12.2%)	1260 (17.0%)	12.6	0.58 (0.48, 0.69)	0.55 (0.46, 0.66)	< 0.001
IMD 2010 quintile							< 0.001^c^
1 (least deprived)	1407 (15.8%)	293 (19.7%)	1114 (15.0%)	20.8	1.00	1.00	
2	1531 (17.2%)	285 (19.1%)	1246 (16.8%)	18.6	0.86 (0.70, 1.04)	0.87 (0.72, 1.07)	0.190
3	1616 (18.2%)	315 (21.2%)	1301 (17.6%)	19.5	0.80 (0.65, 0.98)	0.85 (0.69, 1.05)	0.139
4	2725 (30.6%)	420 (28.2%)	2305 (31.1%)	15.4	0.64 (0.52, 0.79)	0.70 (0.56, 0.87)	0.001
5 (most deprived)	1604 (18.0%)	171 (11.5%)	1433 (19.3%)	10.7	0.46 (0.23, 0.33)	0.52 (0.40, 0.68)	< 0.001
Unknown	19 (0.2%)	5 (0.3%)	14 (0.2%)	26.3			
QRisk2 score (%)	25.2 (5.0)	24.6 (4.5)	25.3 (5.1)		0.86 (0.81, 0.92)^d^	0.82 (0.77, 0.88)^d^	< 0.001
20–24.9%	5357 (60.2%)	968 (65.0%)	4389 (59.2%)	18.1			
25–29.9%	2245 (25.2%)	347 (23.3%)	1898 (25.6%)	15.5			
> 30%	1300 (14.6%)	174 (11.7%)	1126 (15.2%)	13.4			

*OR* odds ratio, *CI* confidence intervals, *IMD* Index of Multiple Deprivation

Data are presented as mean (SD) or *n* (%) unless otherwise stated

^a^All patients invited to participate in the trial from GP practice sites at which it was possible to extract anonymised data

^b^OR per 5 year increase in age

^c^Chi square test for independence

^d^OR per 5% increase in QRisk2 score

As CVD risk increased the odds of response decreased (AOR 0.82 per 5 percentage points; 95% CI 0.77–0.88); for every 5 point increase in QRisk2 score the odds of responding decreased by 18%. Response was lower in patients of black African or Caribbean ethnicity (AOR 0.67; 95% CI 0.45–0.98) and those with missing ethnicity data (AOR 0.55; 95% CI 0.46–0.66) compared to white ethnicity. The odds of response from Asian and other ethnic backgrounds were not significantly different to that of patients of white ethnicity. A median test found the IMD ranks of responders (15,314; 9285–24,774) was significantly higher than non-responders (12,854; 7411–22,330) (*p* < 0.001). The odds of response in the fourth (AOR 0.70; 95% CI 0.56–0.87) and fifth (AOR 0.52; 95% CI 0.40–0.68) most deprived quintiles were significantly lower than the least deprived quartile. Response was higher with increasing age (AOR 1.19 per 5 years; 95% CI 1.12–1.26); the odds of responding increased by 19% for each 5 year increase in age. Odds of responding were higher in male compared to female patients (AOR 1.25; 95% CI 1.08–1.45).

Pairwise comparisons were conducted to explore differences in response between each of the non-white ethnic groups, but there were no significant differences. Response rates of patients with missing ethnicity data were significantly lower than that of patients of south Asian ethnicity (*p* = 0.002). Due to small numbers of invitations to patients of non-white ethnicity, a sensitivity analysis was undertaken to investigate predictors of response to invitation with ethnicity removed from the model. Lower CVD risk (*p* < 0.001), lower levels of deprivation (*p* = 0.001), older age (*p* < 0.001) and male sex (*p* = 0.015) remained significant predictors of response.

## Discussion

In this cross-sectional study of response to an invitation to take part in a RCT of an intensive lifestyle intervention for primary prevention of CVD, we found that likelihood of response reduced with increasing CVD risk in a population who were all at high risk of CVD. Black African or Caribbean patients were less likely to respond than those of white ethnicity, although there were small numbers of black African or Caribbean patients invited. South Asian patients were as likely to respond as those of white ethnicity. We also observed high rates of missing ethnicity data on medical records and this group was less likely to respond compared to patients of white or south Asian ethnicity. Likelihood of response also reduced with increasing deprivation. Older age and male sex predicted greater rates of response.

### Strengths and limitations

The main strengths of the study are the large sample size and the opportunity to assess response bias in a multi-ethnic and socio-economically varied setting, which few lifestyle intervention trials have achieved [12]. Access to anonymised CVD risk and sociodemographic data of large numbers of non-responders, including those who may typically be difficult to reach and unlikely to provide research data, is a unique aspect of this study. As a consequence of this methodology we did not have informed consent to access medical records for further information and so there is a risk of residual confounding by employment status, education and comorbidities [11]. We retrieved data from approximately half of the participating general practices, but as there was no difference in mean general practice level deprivation compared with all other practices in south London it is unlikely this was a significant source of bias. Other studies have reported more detailed data on a smaller number of non-participants, including qualitative feedback on reason for non-participation [11, 15, 24–26], but the current study benefits from an enhanced reach and greater power to demonstrate response biases.

The methodology of recruitment to the MOVE IT trial relied upon general practice database calculations of QRisk2 score, and these calculations were used in the current analysis. We did not seek ethical approval to assess the underlying data used in the QRisk2 calculations. It may be that large proportions of clinical data such as blood pressure, weight and cholesterol:HDL ratio were missing, which has been demonstrated previously [27], in which case they would be replaced with age- and sex-weighted averages. Additionally, where clinical data is present, accuracy of QRisk2 scores may be compromised by the length of time since clinical data had been collected. In future analyses, the extraction of more detailed data from medical records would allow for a sensitivity analysis to explore the effect of missing or outdated data on outcomes.

The use of a 20% QRisk2 score as a screening criteria led to invitations to participate being sent to older, and more male, patients as age and male sex are given a large weighting in the QRisk2 algorithm [6]. Similarly, south Asian ethnicity contributes to an increased QRisk2 score and black African or Caribbean ethnicity to a lower QRisk2 score compared to white ethnicity. Therefore the use of an absolute QRisk2 score as a screening tool in recruitment contributed to limited demographic difference within the target population. Alternative recruitment strategies may include the specification of individual risk factors in the search strategy [28], a relative CVD risk score incorporating a comparison with the average score for an individual of the same age, sex and ethnicity, or a lifetime CVD risk score which tends to identify younger patients [29, 30]. These approaches may assist in identifying patients whose modifiable, rather than non-modifiable, risk factors suggest they would benefit from the intervention as well as increasing the likelihood of a more diverse and representative study sample.

### Interpretation and comparison with other studies

Previous studies have found both higher levels of self-reported health and greater self-reported activity levels [10, 14, 16, 17], as well as lower levels of self-reported health, lower activity levels and a higher CVD risk profile in participants compared to non-participants [11, 13–15]. These contradictory findings may relate to variations in recruitment methodology, the particular target population involved and the aims of the trial. As the current study assessed those who did not respond to an invitation and who are by definition hard to reach, we could not compare self-reported physical activity or health problems in responders and non-responders, but had the advantage of reporting a standardised CVD risk algorithm score. Already feeling healthy is a frequently cited cause of non-participation [11, 15, 24–26], but using a QRisk2 cutoff score of 20% in the current study meant that all those invited to participate would be considered at high risk of CVD [31], a fact which was communicated to patients in the invitation letter. Those patients at the highest risk level may not respond due to a number of reasons which could be explored through qualitative work.

Previous studies have found greater response rates in those of white ethnic background and those residing in more affluent areas [11, 12], but the generalisability of available data is limited due to the majority of participants in previous trials being white, middle-aged females, and the lack of information on ethnicity and deprivation available in published trials [32]. Our findings contribute to concerns that research in general fails to reach socially disadvantaged groups [33]. However, the trial did reach patients of south Asian ethnic backgrounds as much as patients of white ethnic backgrounds, which is a promising finding given higher CVD risk in this group [34].

Ability to undertake an analysis of anonymised primary care data is limited by the large amount of missing ethnicity data on general practice databases, and this impacted the data collected for the current study. Providing self-report ethnicity data has been found to be less likely in ethnic minority populations in the United States [35], and ethnic minority patients are less likely to provide ethnicity information to health care providers due to concerns over how the information may be used [36]. In a study of hospital patients with cancer in England, there were only small differences in proportion of missing ethnicity data recorded in secondary care between self-report ethnic backgrounds, and much larger differences were found between different hospitals [37]. The missing ethnicity data in primary care may be related to either general practice ethnicity-recording processes or patient reluctance to divulge information, and further research is needed to explore this and which patient groups are more likely to have missing ethnicity data. Increasing ethnicity recording in primary care is vital for understanding the representativeness of study samples. Patients reluctant to provide optional self-report ethnicity data in primary care may similarly be at reduced likelihood of responding to a RCT invitation, as our findings may allude to. Increasing awareness in certain subgroups of the population through varied approaches to recruitment, such as employing telephone or email reminders for non-responders [38], or the assistance of a recruitment mediator who is a member of the subgroup [39], could help to improve study representativeness amongst ethnic minority and less affluent groups.

The majority of those invited to the trial were above working age, and likelihood of response increased with age. Burden of time is a common reason for non-participation in lifestyle interventions [11, 24–26], and older invitees are more likely to be retired and may have more flexibility to participate. Other studies have found that participation rate increases with age in working age populations [15], but reduces into older age possibly as patients become more frail [11, 16]. Tailoring a lifestyle intervention for primary prevention of CVD may necessitate changing the setting and target population from primary care to the workplace, and making the intervention more desirable by reducing time burden, increasing flexibility and using digital technologies.

It has previously been reported that females are more likely to participate in lifestyle intervention trials [11], however reviews of the literature indicate many lifestyle intervention trials have recruited female only samples [12, 32]. The higher response rate of males in the current study, secondary to the larger proportion of males invited, may reflect public opinion that CVD is a predominantly male disease despite also being the leading cause of death in females [5, 40]. Previous studies assessing participation in trials of those who already have CVD [41, 42], and in a survey regarding cardiovascular risk factors [43], also found lower response in females. In a small sample of those providing feedback on the reasons for non-participation, females were more likely to mention caring responsibilities as a barrier [41]. Increasing public awareness of CVD risk in females, as well as providing flexible appointments as previously mentioned, may be required to increase female participation.

The findings of this study are specific to the RCT under study, and should not be conflated with likelihood of response to similar interventions in clinical practice. Reasons for non-response may be research-specific, or in combination with lack of motivation or interest in the particular intervention. As participants were not contacted for this analysis, we could not explore patient perspectives on study-specific materials such as the invitation letter sent by the patient’s general practice. Further research is required to gain a greater understanding of the influence of study-specific invitation procedures, patient perspectives of research in general and willingness to undergo an intervention.

## Conclusions

We have demonstrated that it is possible to ethically assess response bias to a RCT with the use of anonymised patient data. As a result, we have highlighted the risk that RCTs of lifestyle interventions may fail to recruit the highest risk patients, ethnic minority patients and those residing in more socioeconomically deprived areas, which could result in implementation of interventions which increase health inequalities. Such analyses are limited by missing ethnicity data on primary care databases. Improvements in ethnicity reporting would aid understanding of whether RCTs have successfully recruited a representative sample. Future RCTs of lifestyle interventions should aim to proactively minimise recruitment biases and report on the representativeness of their samples as part of a process evaluation.

## Additional files

Additional file 1: Letter of invitation to participants. (DOC 35 kb)

Additional file 2: Participant information sheet. (DOC 1083 kb)

## Abbreviations

AOR: Adjusted odds ratio
CBT: Cognitive behaviour therapy
CCG: Clinical Commissioning Groups
CI: Confidence interval
CVD: Cardiovascular disease
IMD: Index of Multiple Deprivation
LDL: Low density lipoprotein
LSOA: Lower layer super output area
MI: Motivational interviewing
MOVE IT: MOtiVational intErviewing InTervention
OR: Odds ratio
RCT: Randomised controlled trial
SD: Standard deviation
UK: United Kingdom
