# Supplementary material for: Response bias to a randomised controlled trial of a lifestyle intervention in people at high risk of cardiovascular disease: a cross-sectional analysis
Source: BMC Public Health. 2018 Sep 4;18:1092. doi: 10.1186/s12889-018-5939-y (PMC6124010; doi:10.1186/s12889-018-5939-y)
Supplement: Supplementary file 1 — Letter of invitation to participants. (DOC 35 kb) [file 12889_2018_5939_MOESM1_ESM.doc]

GP practice headed paper

Dear Participants name

**RE: A multi-centre randomised controlled trial comparing the effectiveness of enhanced motivational interviewing with usual care for reducing cardiovascular risk**

We would like to invite you to take part in an NHS funded research study on heart disease that we are taking part in. You are being asked to take part because our records suggest that there is over 20% chance you may develop heart disease in the next ten years.

The study is being carried out by a team of doctors and clinical researchers from King’s College London and St George’s University London. We are finding out whether a programme which provides support around changing lifestyles, such as diet and physical activity, can reduce the risk of developing heart disease.

Heart disease can be prevented, yet we know that people often have difficulty in changing their diet, increasing their physical activity, quitting smoking and taking medication. Motivational interviewing can help people identify barriers stopping them making lifestyle changes and encourage them to be more confident about making changes.

In this study we would like to find out if motivational interviewing given by healthy lifestyle facilitators can help patients make healthier lifestyle changes and whether these changes can be kept up for 2 years.

If you agree to take part you will be placed into **one of three** possible categories:

1) **Usual GP care,**

**2) Individual motivational interviewing sessions with the healthy lifestyle facilitator**

**3) Group motivational interviewing sessions with the healthy lifestyle facilitator**

The **study will take place at your GP practice or local facility**.

If you would like to know more about this study, please read the information sheet attached. If after reading this you are interested in the study and would like to find out more about taking part you can either:

1. Complete the attached form and return it using the addressed envelope provided. This lets us know that you are interested in finding out more and gives your permission to be contacted by a member of the research team for a discussion.

Or

2. Contact the research team directly:

Name of researcher and contact details

Email Address

Yours sincerely,

General Practitioner

-----------------------------------------------------------------------------------------------------------------

*If you would like to find out more about this study please fill out your details below and then tear off this slip and return it using the addressed envelope enclosed.*

I am interested in finding out more and give permission for a member of the research team to contact me

Name: ______________________________________

Address: ______________________________________

______________________________________

______________________________________

______________________________________

Phone number: _____________________________________

E-mail address: _____________________________________

Name of GP practice: ________________________________
