# Supplementary material for: Response bias to a randomised controlled trial of a lifestyle intervention in people at high risk of cardiovascular disease: a cross-sectional analysis
Source: BMC Public Health. 2018 Sep 4;18:1092. doi: 10.1186/s12889-018-5939-y (PMC6124010; doi:10.1186/s12889-018-5939-y)
Supplement: Supplementary file 2 — Participant information sheet. (DOC 1083 kb) [file 12889_2018_5939_MOESM2_ESM.doc]

## Institute of Psychiatry

## Department Of Psychological Medicine

King’s College London

10 Cutcombe Road

London

SE5 9RJ


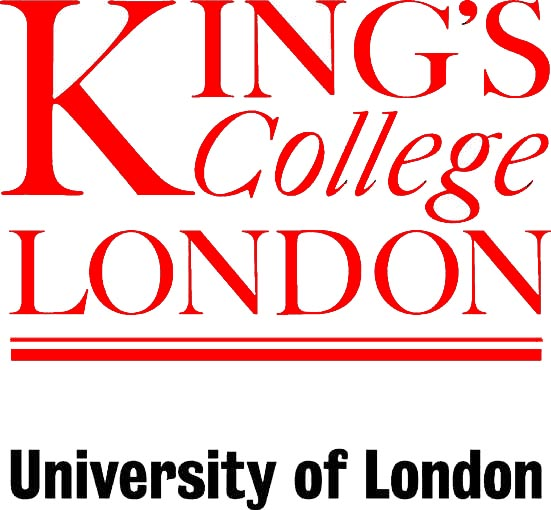


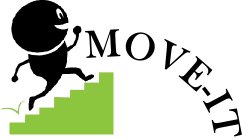


**Participant information sheet**

**The MOVE-IT study**: *A multi-centre randomised controlled trial comparing the effectiveness of enhanced motivational interviewing with usual care for reducing cardiovascular risk.*

You are being invited to take part in an NHS funded research study being carried out by Kings College London and St. Georges Hospital.

**Before you decide whether or not you wish to take part, you should read the information provided below carefully and, if you wish, discuss it with your family, friends or GP. If you would like any more information, have any questions or something is not clear, please feel free to contact the research team, details are at the end of this letter.**

You will be given this information sheet and a signed consent form to keep if you decide to take part in the study.

**Why am I being asked to take part?**

Your medical records suggest that there is over a 20% chance that you may develop heart disease in the next ten years. We would like to see if we could help you reduce this risk, so we are inviting you to take part in this research study.

**Purpose of the study**

Motivational interviewing (MI) is a therapy which involves talking about your thoughts and feelings to help you make positive changes to your life. This will be provided by a healthy lifestyle facilitator who aims to help people become more confident, willing, and committed to making changes to their lifestyle. There is evidence to suggest that MI helps people make short term lifestyle changes, we want to learn if these changes can be kept up in the long-term. We would also like to understand the differences between being seen individually or in a group. We would like to test whether motivational interviewing with additional coping skills can help people at high risk of developing heart disease make healthier lifestyle choices and stick to these over the course of two years.

Factors which may increase your chances of developing heart disease include cigarette smoking, poor diet and physical inactivity. If these lifestyle choices continue long-term this can increase the risk of other serious health problems, such as, high blood pressure, weight gain, stroke, and diabetes. This study will be looking at whether motivational interviewing, as described above, can help improve diet and physical activity, to reduce the risk of developing heart disease. Even though most of us are aware that we should be eating healthier and be more active, for many people it can be very difficult to achieve this in the long term for different reasons.

**Do I have to take part?**

No. It is up to you to decide. We will describe the study and go through this information sheet, which we will then give to you. If you would like to take part we will ask you to sign a consent form to show you have agreed to take part. You are free to withdraw at any time, even after you have agreed to be contacted, without giving a reason. This would not affect the standard of care you receive.

**What will happen to me if I take part?**

If you are happy to participate, one of the researchers would like to meet with you and take some measurements. This appointment would last approximately one hour and will involve:

- Measuring your height, weight and blood pressure.
- Taking a small sample (about 10 mls or two spoonfuls) of blood from your arm to check your cholesterol and blood glucose.
- Answering some questions about your health.
- At the end of the appointment you will be given a small pedometer to wear on your hip for 7 days, and then post back.

You will then be randomly placed into one of three categories. You will have an equal chance of being in any of the categories. The categories are:

1. Usual care

- If you are placed in this category you will not attend the motivational interviewing sessions, but will continue to see your GP as normal.

If you are placed in the individual or group categories you will be given an appointment to see a healthy lifestyle facilitator. The healthy lifestyle facilitators are specially trained in motivational interviewing and will deliver the sessions at your GP practice or a local community venue.

2. Individual motivational sessions

- During the study you will receive 10 therapy sessions over 12 months, with each session lasting 30 minutes. At first there are 6 weekly sessions and then one session at 3, 6, 9 and 12 months.

3. Group motivational sessions

- During the study you will receive 10 therapy sessions over 12 months, with each session lasting 90 minutes. At first there are 6 weekly sessions and then one session at 3, 6, 9 and 12 months.

Everybody will continue to have their usual care from their GP. Everybody will meet with the researchers after 1 and 2 years to repeat the measurements taken in the first appointment.

The aim of this study is to understand how useful motivational interviewing is in reducing the risk of heart disease. To help us understand what happens during the therapy sessions we would like to audio record some of these. Any sessions that are taped will be written down and your identity will be kept anonymous throughout this process. During the course of the study we will contact you to discuss your thoughts and experiences of the therapy that you have received.

**What are the possible disadvantages or risks of taking part?**

We do not expect there to be any disadvantages of participating in the study. The therapy is based on eating more healthily, increasing physical activity, and gradual and sustainable weight loss. As we would request permission for a blood sample this may cause some discomfort.

**What are the possible advantages of taking part?**

We cannot promise the study will help you, but you may receive motivational interviewing. There are potential health benefits for participants in the study, as it has been shown taking part in research usually is related to better outcomes. The results of our research could also help to develop new treatments for people at risk of heart disease.

**Will my information be kept confidential?**

Yes. We follow ethical and legal practice and all information about you will be handled in confidence. The data you give us will be made anonymous.

**What will happen to the results of the research study?**

The research data will be stored in a locked filing cabinet and in a locked room. Patients can only be identified by a unique and anonymous study number. Electronic data will be password protected and any direct quotations will be anonymised.

**What will happen to my collected data if I withdraw from the study?**

If you decide to leave the study any research data and blood previously collected with your agreement would be kept and used in the study. We would not collect any more information from you or expect you to take part in any other research activities.

**Who is funding the research?**

The National Institute for Health Research (NIHR) Health Technology Assessment (HTA) is funding this study. The NIHR is the organisation which conducts research for the NHS. Any of your travel expenses for the study may be refunded.

**Who has reviewed the study?**

The research proposal has been reviewed by staff in the Academic Department of Psychological Medicine at King’s College London and by the local Research Ethics Committee at Dulwich, London.

**Future research**

We have funding for this research for 4 years in total but would like to continue collecting information about your health for an additional 5 years if you agree. This is to see if the motivational interviewing is helpful in the long-term. We would like to request your permission to continue to collect health data from your medical notes and to contact you after the 4 year study is over.

We would like to retain your blood samples for up to 5 years after the study ends for further analysis.

We would also like to invite a number of people for further interviews to tell us about their experience of the study.

You can record your decision about these on the consent form.

**Contact for further information**

If you have any questions or require further information about this study now or at any time during the study, you are very welcome to get in touch with the research team on Tel. 020 7848 5759/5785.

If you have any questions concerning your rights as a study participant you may wish to read the following leaflet: Getting Involved in Research: A guide for consumers, available at:-

<http://www.invo.org.uk/pdfs/guide_for_consumers.pdf> or contact the Consumers in NHS Research Support Unit, Tel: 01962 872247.
